# Antibiotic Class-Specific Effects on Inflammatory Bowel Disease: Microbiome Disruption, Risk, and Recovery

**DOI:** 10.3390/ijms27146502

**Published:** 2026-07-22

**Authors:** Bhargavi Rajarathinam, Pranav V. Nair, Neeraja Murali, Ganga Lekshmi, Abitha K. Sajeev, Archa B. Pillai, Anita Thomas, Sreetha Hely, Kalyani Arun, Vidhya Prakash, Bipin G. Nair, Parvathy Venugopal, Rajaguru Aradhya

**Affiliations:** School of Biotechnology, Amrita Vishwa Vidyapeetham, Kollam 690525, Kerala, India; bagi.eutopia@gmail.com (B.R.); pranavvnair2005@gmail.com (P.V.N.); neerajamwol@gmail.com (N.M.); gangalekshmi613@gmail.com (G.L.); abithaksajeev01@gmail.com (A.K.S.); archab2004@gmail.com (A.B.P.); anitaminithomas313@gmail.com (A.T.); sreethah@am.amrita.edu (S.H.); kalyaniarun147@gmail.com (K.A.); vidhyaprakash@am.amrita.edu (V.P.); bipin@am.amrita.edu (B.G.N.)

**Keywords:** antibiotic exposure, inflammatory bowel disease (IBD), gut microbiome, dysbiosis, fecal microbiota transplantation, antibiotic stewardship, microbiome-targeted therapies

## Abstract

Inflammatory bowel disease (IBD), including Crohn’s disease (CD) and ulcerative colitis (UC), are chronic inflammatory diseases resulting from complex interactions between host genetics, environmental factors, immune dysregulation, and the gut microbiome. Among environmental exposures, antibiotics have emerged as important factors of IBD risk and disease course because of their profound effects on intestinal microbial communities. This review synthesizes current evidence on the class-specific effects of antibiotics on IBD, integrating epidemiological, mechanistic, and clinical studies to examine how different antibiotic classes influence disease susceptibility, progression, and microbiome recovery. Current evidence indicates that antibiotic-associated IBD risk varies according to antibiotic class, cumulative exposure, age at exposure, and antimicrobial spectrum, with broad-spectrum and anti-anaerobic agents showing the strongest associations. Mechanistically, antibiotics promote dysbiosis by depleting beneficial commensal bacteria, disrupting microbial metabolite production, expanding pathobionts and the intestinal resistome, and impairing epithelial barrier integrity and immune homeostasis. The review also discusses microbiome-preserving and microbiome-restorative approaches, including antimicrobial stewardship, fecal microbiota transplantation, prebiotics, probiotics, synbiotics, postbiotics, and dietary interventions, as potential strategies to mitigate antibiotic-associated dysbiosis. Overall, the evidence highlights the class-specific effects of antibiotics in IBD and underscores the importance of microbiome-informed antimicrobial stewardship and precision therapeutic strategies to optimize patient outcomes while minimizing long-term disruptions of host–microbiome homeostasis.

## 1. Introduction

Inflammatory bowel disease (IBD) is a chronic inflammatory disease of the gastrointestinal tract defined by chronic and relapsing intestinal inflammation [1]. Clinical manifestations are abdominal pain, diarrhoea, rectal bleeding, weight loss, and the influx of neutrophils and macrophages that produce cytokines, proteolytic enzymes, and free radicals that result in inflammation and ulceration [2]. IBD is primarily divided into two subtypes based on the disease location, clinical manifestations, and histopathological characteristics: ulcerative colitis (UC) and Crohn’s disease (CD). CD can affect any segment of the gastrointestinal tract, from the oral cavity to the anus, although individual patients commonly exhibit involvement of a specific region, such as the small intestine or colon. CD is often marked by discontinuous, transmural inflammation with focal lesions that may progress to deep ulcerations that can perforate the bowel wall, leading to complications such as fistula formation, abscesses, and fibrotic strictures. In contrast, UC is marked by continuous mucosal inflammation confined to the colon that usually starts in the rectum and extends proximally in a circumferential and uninterrupted pattern [3]. Several causal factors have been found to be involved in the pathogenesis of IBD, but none are universally present in all patients [1]. Several studies have identified a major role of both genetic and environmental factors in the pathogenesis of IBD. Genome-wide studies have found over 200 genetic loci associated with IBD, implicating key immunological pathways such as innate immunity (e.g., *NOD2*), immune regulation (e.g., *IL23R*), and autophagy (e.g., *ATG16L1*) [4,5,6]. Recent studies have shown that the microbiota is primarily shaped by host factors linked to industrialization, including body mass index, glycemic response, high-density lipoprotein cholesterol, and lactose consumption [7]. Environmental factors, such as smoking, diet, medications (specifically antibiotics), circadian rhythm, and stress, have also been studied in relation to the development of IBD. Among environmental factors, antibiotics have garnered particular attention. Antibiotics are often used in IBD treatment to both reduce the overall bacterial concentrations in the gut lumen and modify the composition of the gut microbiome in favor of beneficial bacteria. Ciprofloxacin, aminoglycosides, or rifaximin are some of the antibiotics routinely prescribed to IBD patients to target specific bacteria. Although antibiotics remain valuable adjunctive therapies in selected IBD settings, accumulating evidence suggests that their widespread use may simultaneously increase disease susceptibility through microbiome disruption. Multiple meta-analyses and large cohort studies now report that antibiotic exposure is associated with heightened risk of both pediatric and adult-onset IBD with a dose–response effect and variation by antibiotic class [8,9]. Early childhood antibiotic exposure has been linked to an increased risk of IBD [10]. For example, pooled data from a meta-analysis show that children (age < 18) exposed to antibiotics have roughly a 1.4–1.6-fold higher risk of later developing CD compared to unexposed children [9]. In a recent Danish study, early-life antibiotic use nearly doubled the odds of pediatric IBD. Likewise, broad national cohort data indicate that antibiotic use in adults is linked to increased IBD incidence, with a clear dose–response (more courses → higher risk) and a time-lagged effect peaking 1–2 years after exposure [8]. However, these epidemiologic associations must be interpreted cautiously, as reverse causation and confounding are important concerns: individuals predisposed to IBD often suffer more infections and thus receive more antibiotics making it difficult to distinguish whether antibiotic exposure directly contributes to disease pathogenesis or merely reflects preclinical disease activity.

Importantly, these risk associations are antibiotic-class specific. Nearly all antibacterial classes have been implicated in raising IBD risk (except nitrofurantoin), but the strongest associations are seen for agents targeting anaerobic and Gram-negative gut bacteria. In particular, nitroimidazole antibiotics (e.g., metronidazole) and fluoroquinolones have consistently shown higher risk estimates, whereas narrow-spectrum or non-GI-targeting drugs (e.g., nitrofurantoin) appear neutral [8,11]. These class-specific patterns suggest that spectrum and ecological impact matter: broad-spectrum antibiotics and those that deplete anaerobes likely drive greater dysbiosis of the gut ecosystem.

Antibiotic treatments significantly alter the gut microbiome. Short courses of broad-spectrum antibiotics are well documented to reduce microbial diversity and eradicate many beneficial anaerobic commensals (including short-chain-fatty-acid producers that support barrier integrity) [3,12,13]. Such perturbations often permit overgrowth of facultative pathogens (e.g., Enterobacteriaceae) and expansion of the gut resistome (antibiotic-resistance gene pool) [14,15]. In other words, antibiotic use can transform the gut microbiome into a less resilient, more inflammatory community–a state plausibly conducive to triggering IBD in genetically susceptible hosts. Indeed, genetic studies of IBD (e.g., *NOD2*, *IL-23* pathway) point to impaired handling of commensal microbes as a key pathogenic mechanism [16]. Thus, by disrupting normal host–microbe symbiosis, antibiotics may inadvertently promote the dysbiosis and immune dysregulation thought to underlie IBD.

The scope of this review is to synthesize current evidence specifically on how different classes of antibiotics affect IBD risk and course, mediated through the gut microbiome. We focus on class-specific epidemiology of IBD incidence and flares, the dual roles of antibiotics as both therapies and pathogenic triggers, and emerging strategies to mitigate microbiome damage (e.g., targeted probiotics, dietary approaches, phage therapy). In contrast to prior reviews that mention antibiotics as part of broader microbiome discussions, we explicitly highlight antibiotics’ unique immunological and resistome-related impacts in IBD. This review thus provides a novel class-based perspective, integrating epidemiology, mechanistic microbiome studies, and translational interventions in one place.

## 2. Antibiotic Use in Inflammatory Bowel Disease: Therapeutic Applications and Disease Risk

### 2.1. Therapeutic Paradox

Antibiotics have a complex and context-dependent role in the management of IBD and are most commonly used in CD, particularly in patients with colonic involvement, perianal disease, abscesses, or fistulas. However, they are most often used for adjunctive therapy in IBD and are not recommended as routine first-line therapies. Their clinical benefit is mainly attributed to the reduction in bacterial translocation, suppression of pathogenic bacteria and modulation of intestinal microbial communities that contribute to mucosal inflammation and altering the composition of the intestinal microbiota to promote beneficial bacteria [17]. Medications for IBD may target specific bacteria, such as ciprofloxacin, aminoglycosides, or rifaximin for Gram-negative enteric bacteria, metronidazole for anaerobes (specifically *Bacteroides fragilis*), or antitubercular medications to treat mycobacterial infection, Mycobacterium avium subspecies paratuberculosis (MAP), which has been linked to CD development [18].

**Figure 1:**
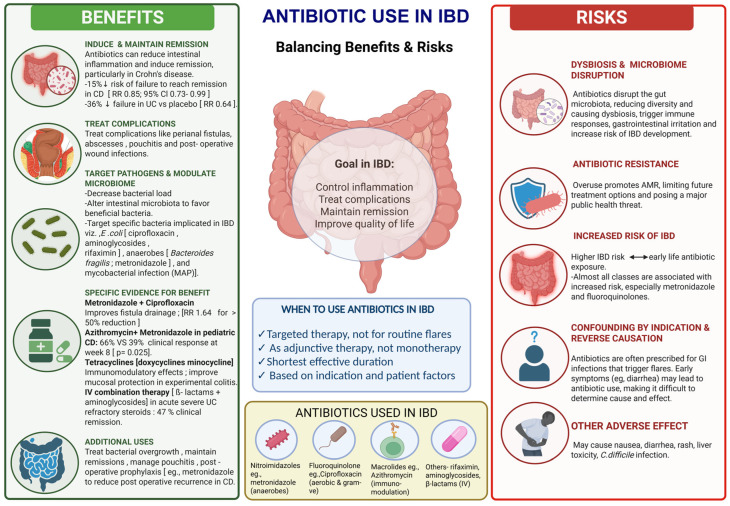
**Antibiotic Exposure in Inflammatory Bowel Disease: Adjunctive Therapeutic Applications and Microbiome-Associated Risks.** Antibiotics provide therapeutic benefits by reducing pathogenic bacterial burden, treating infectious complications such as abscesses and pouchitis, and modulating the gut microbiota to alleviate intestinal inflammation. On the other hand, repeated or inappropriate antibiotic exposure can disrupt the gut microbial ecosystem, leading to depletion of beneficial commensal bacteria, reduced microbial diversity, altered microbial metabolite production, expansion of pathobionts and the intestinal resistome, impaired epithelial barrier function, and immune dysregulation. These antibiotic-induced alterations may increase susceptibility to IBD development, contribute to disease progression, and promote recurrent intestinal inflammation. Created in BioRender. Nair, B. (2026) https://BioRender.com/nz237dq (accessed on 20 May 2026).

Antibiotics in IBD are mainly used to treat the primary disease process (including luminal disease and fistulizing disease for CD and colitis in the case of UC as well as to treat bacterial overgrowth and septic complications of IBD, such as abscesses and postoperative wound infections. Additionally, it can be used to treat pouchitis or sustain remissions [19]. Targeted antibiotic therapy is used in IBD to rebalance the gut microbiome and treat complications such as perianal fistulas and abscesses, rather than for routine flare-ups. Antibiotics may reduce CD activity and help achieve remission [19] (Figure 1). Antibiotics such as ciprofloxacin, metronidazole, azithromycin, and rifaximin may play a limited role in mild to moderate luminal CD. Metronidazole has been shown to be beneficial in preventing postoperative recurrence in CD, but its long-term benefits are still unknown. Even more debatable is the use of antibiotics in UC, although research employing broad-spectrum oral antibiotic cocktails has suggested a potential role in both acute severe colitis and chronic persistent UC [20]. Clarithromycin-based or anti-MAP regimens show remission or response in refractory CD, but results are inconsistent across trials, and no robust, generalizable odds ratio has emerged, indicating only modest and heterogeneous benefit [19]. Antibiotics are also frequently prescribed for GI infections, which may themselves contribute to or trigger IBD flares. Early symptoms of IBD flares (e.g., diarrhoea) may call for antibiotic use. These scenarios introduce the possibility of confounding by indication and reverse causation [21]. Conversely, antibiotic exposure may act as an environmental factor contributing to IBD pathogenesis. Almost all types of antibiotics were associated with an increased IBD risk, and the degree of risk varied between antibiotic classes, for example, the use of metronidazole and quinolones was associated with relatively higher risk estimates. Recent studies indicate that antibiotic usage can significantly disrupt the gut microbiota, leading to microbial imbalance, or dysbiosis.

Antibiotic use has been identified as a risk factor for the development of IBD in children. A Danish national cohort study found that early antibiotic use nearly doubled the risk of developing IBD in children. This risk was primarily linked to a higher risk of CD compared to UC and was most prominent during the initial months of use. A nationwide case–control study in Sweden found that antibiotic use nearly doubled the risk of developing IBD [22]. Subgroup analysis found that older adults who had received two or more courses of antibiotics were more likely to develop IBD [23].

### 2.2. Epidemiological Framework of Antibiotic-Induced Risk and Key Confounders

Large-scale, population-based cohort studies and national registry datasets have firmly established an association between exposure to antibiotics and the development of IBD. Recent evidence indicates that the risk of IBD varies by antibiotic class, spectrum of activity, and duration of exposure. These differences reflect the different effects of antibiotics on gut microbial composition, diversity, and function [22]. Several large-scale population-based studies show a positive association between antibiotic use (especially broad-spectrum antibiotics) and the risk of new-onset IBD, indicating that antibiotics may facilitate the development of the disease by altering the gut microbiota [23]. For example, a 2025 meta-analysis of 153,000 IBD patients found that most antibiotic classes were linked to higher new-onset IBD risk, with metronidazole (OR ≈ 1.70) and fluoroquinolones (OR ≈ 1.56) conferring particularly high risk [11].

#### 2.2.1. Age-Specific Associations

The association between antibiotic exposure and inflammatory bowel disease (IBD) varies across the lifespan, reflecting differences in microbiome development, immune maturation, and microbial resilience. Although antibiotic-associated IBD risk has been demonstrated in pediatric, adult, and elderly populations, accumulating evidence suggests that early-life exposure represents a particularly vulnerable period during which disruption of the developing gut microbiome may have long-lasting immunological consequences.

##### Pediatric and Early-Life IBD Risks

The neonatal and early childhood periods are characterized by rapid microbial colonization and maturation of the intestinal immune system. During this critical developmental window, the gut microbiota establishes essential host–microbe interactions that regulate epithelial barrier integrity, immune tolerance, and metabolic homeostasis [24]. Antibiotic exposure during infancy can interrupt these processes by reducing microbial diversity, delaying colonization by beneficial anaerobes such as *Bifidobacterium* and *Faecalibacterium*, and promoting expansion of opportunistic pathobionts, resulting in persistent alterations in microbial composition and function [25,26]. Early-life gut dysbiosis from antibiotic uptake has long-term effects on innate and adaptive immune cell maturation, making infants prone to IBD, leaky gut, celiac disease, and colorectal cancer [27,28]. Multiple epidemiological studies have identified early-life antibiotic exposure as an independent risk factor for pediatric IBD. A Danish national cohort study found that early antibiotic use nearly doubled the risk of developing IBD in children [29]. This risk was primarily linked to a higher risk of CD compared to UC and was most prominent during the initial months of use. A systematic review and meta-analysis by Nguyen et al. demonstrated that antibiotic exposure during childhood was associated with a significantly increased risk of subsequent IBD, with the association being stronger for Crohn’s disease than ulcerative colitis [23]. Similarly, Shaw et al. reported that in pediatric IBD patients, 58% had one or more antibiotic dispensations in their first year of life compared with 39% of controls. Repeated antibiotic exposure during infancy and early childhood was associated with a dose-dependent increase in pediatric-onset IBD, supporting the hypothesis that cumulative microbiome disruption during immune development contributes to disease susceptibility [30].

Mechanistically, early antibiotic exposure may impair the establishment of regulatory immune networks by reducing microbial-derived short-chain fatty acids, altering bile acid metabolism, and limiting microbial stimulation required for normal regulatory T-cell differentiation. These alterations may result in persistent immune dysregulation that predisposes genetically susceptible individuals to chronic intestinal inflammation later in life [31,32].

##### Adult-Onset IBD Risk

Although the developing microbiome appears particularly susceptible to antibiotic perturbation, recent evidence indicates that antibiotic-associated IBD risk is not restricted to childhood. A nationwide Danish population-based cohort study including more than six million individuals demonstrated that antibiotic exposure was associated with an increased incidence of IBD across all age groups, Furthermore, each additional antibiotic course conferred progressively greater risk, highlighting a consistent dose–response relationship independent of age [22].

Overall, current evidence suggests that age influences the magnitude rather than the existence of antibiotic-associated IBD risk. Early-life exposure may produce long-lasting alterations in immune programming through disruption of microbiome development, whereas in adults repeated or broad-spectrum antibiotic exposure appears to overcome microbial resilience, leading to persistent dysbiosis and increased susceptibility to intestinal inflammation. These findings underscore the importance of judicious antimicrobial prescribing throughout life while emphasizing particular caution during the critical early-life window.

#### 2.2.2. Broad-Spectrum vs. Narrow-Spectrum Antibiotics

Broad-spectrum antibiotics, particularly those targeting anaerobic bacteria, have been consistently associated with a higher risk of IBD. Multiple population-based studies have implicated classes such as fluoroquinolones, β-lactam antibiotics (including penicillin and cephalosporins), and macrolides. These agents induce prolonged alterations in gut microbiota, including depletion of key commensal taxa such as *Firmicutes* and *Bacteroidetes*, which are essential for maintaining intestinal homeostasis [33,34]. In contrast, narrow-spectrum antibiotics appear to have a comparatively lower impact on microbiome structure and a weaker association with IBD risk, although data remain limited and sometimes inconsistent.

Broad-spectrum antibiotics had higher incidence rate ratios (IRRs) than narrow-spectrum antibiotics even 4–6 weeks after use. Short-term risk increases were observed for several classes, including quinolones, penicillin, nitroimidazoles, and macrolides. Broad-spectrum antibiotics had a greater impact on IBD flare risk than narrow-spectrum agents, possibly due to more widespread disruption of commensal microbial communities, resulting in more severe dysbiosis [35]. Oral formulations and broad-spectrum antibiotics were linked to a higher short-term incidence of IBD flare-ups, while injectable or narrow-spectrum antibiotics showed no such association [21].

#### 2.2.3. Dose–Response Effects, Duration of Exposure, and Confounding Factors

A dose–response relationship between antibiotic exposure and IBD, with greater cumulative exposure (number of prescriptions, duration of therapy, or diversity of antibiotic classes) associated with increasing disease risk, is one consistent and biologically plausible feature of the association. Large population-based cohort studies show that individuals over 40 have a higher risk of IBD due to antibiotic exposure. Antibiotics targeting gastrointestinal pathogens pose a higher risk after 1–2 years of exposure [22]. Antibiotic use during childhood nearly doubled the risk of developing IBD. CD patients posed a higher risk than those with UC [23].

Faye et al. (2023) [22] found an association between antibiotic exposure and subsequent development of IBD in a nationwide population-based cohort of individuals aged ≥10 years. IBD incidence was elevated in all age groups as compared to participants with no previous use of antibiotics, including 10–40 years (IRR 1.28, 95% CI: 1.25–1.32), 40–60 years (IRR 1.48, 95% CI: 1.43–1.54), and ≥60 years (IRR 1.47, 95% CI: 1.42–1.53). As cumulative antibiotic exposure increased, so did the risk, indicating a dose-dependent relationship. In those aged 10–40 years, the IRR of each additional antibiotic course was 1.11 (95% CI: 1.10–1.12), 1.15 (95% CI: 1.14–1.16) in those aged 40–60 years, and 1.14 (95% CI: 1.13–1.15) in those aged ≥60. Exposure to five or more antibiotic courses resulted in the strongest associations, with IRRs of 1.69 (95% CI: 1.61 to 1.76), 2.12 (95% CI: 2.01 to 2.23), and 1.95 (95% CI: 1.85 to 2.04) in the 10 to 40 years, 40 to 60 years, and ≥60 years age groups, respectively. Temporal analyses revealed that risk estimates were highest within the first 1–2 years of antibiotic exposure, and then gradually decreased across all age groups [22]. Another meta-analysis found that the risk of IBD increased with longer antibiotic exposure and higher prescription frequency. Patients who received ≥3 courses of antibiotics were significantly more susceptible [36].

Mechanistically, repeated or prolonged antibiotic exposure may lead to cumulative loss of microbial diversity, depletion of keystone commensals, and expansion of pathobionts, thereby promoting immune dysregulation and impaired mucosal barrier function. However, interpretation of these associations is complicated by indication bias and confounding factors, which remain major challenges in observational studies. Antibiotics are often prescribed for infections—particularly gastrointestinal or respiratory infections—that may themselves influence IBD risk. For example, enteric infections can induce mucosal inflammation, alter microbial composition, and potentially initiate immune responses that predispose to chronic intestinal inflammation. This raises the possibility of reverse causation, where early subclinical manifestations of IBD (e.g., mild gastrointestinal symptoms) lead to increased antibiotic use before formal diagnosis.

##### Reverse Causation and Diagnostic Lag-Periods

The prodromal phase of both Crohn’s disease (CD) and ulcerative colitis (UC) is often characterized by non-specific gastrointestinal symptoms—such as intermittent abdominal discomfort, altered bowel habits, or occult bleeding which precede formal IBD diagnosis by months or years. These early symptoms are often misattributed to acute infective gastroenteritis or bacterial infections, prompting inappropriate antibiotic use. To isolate this confounding spike in prescribing from a true causal etiology, contemporary epidemiological designs utilize strict diagnostic lag-periods [22]. Population data show that when antibiotic exposures within 1 to 2 years prior to the index diagnosis date are artificially excluded, the observed risk elevations are attenuated but remain statistically robust [22,23]. This persistence confirms that independent of prodromal misprescribing, true antibiotic-mediated pathophysiological risks exist [23,29].

Recent large-scale studies and meta-analyses have attempted to address these limitations through adjustment for infection-related variables and stratified analyses. While such adjustments attenuate the strength of association, significant dose-dependent relationships persist, supporting a potential causal contribution of antibiotics independent of underlying infections [11]. Nonetheless, residual confounding cannot be fully excluded, and emerging models propose a “multi-hit” framework, in which antibiotic exposure interacts with host genetics, environmental triggers, and prior infections to drive disease onset.

Collectively, current evidence supports a model in which both the intensity and timing of antibiotic exposure are critical determinants of IBD risk, while also emphasizing the need for cautious interpretation due to inherent confounding in epidemiological data. Future longitudinal studies integrating microbiome, clinical, and environmental data will be essential to disentangle causality and refine risk stratification.

### 2.3. Antibiotic Class-Specific Risk Associations with IBD

Large cohort and case–control studies have identified differential risks associated with specific antibiotic classes. Broad-spectrum antibiotics that significantly disrupt gut anaerobes were found to contribute the highest risk, whereas agents with minimal gut impact show little or no effect. Table 1 compares major classes on mechanism, typical uses, IBD incidence risk. The following sections summarize each class.

#### 2.3.1. High-Risk Broad-Spectrum Antibiotics

##### Fluoroquinolones

Fluoroquinolones are broad-spectrum antibiotics. They are proven effective against both aerobic Gram-positive and Gram-negative organisms [37] by inhibiting the type II DNA topoisomerases (gyrases), essential for bacterial mRNA transcription and DNA duplication [38]. Faye et al. (2023) [22] showed that fluoroquinolones have the highest risk of developing IBD. The risk was highest among persons of 40 years and older [22]. In children, an older meta-analysis reported particularly high HR of 3.70 for Crohn’s disease after fluoroquinolone use [10].

Mechanistically, fluoroquinolones induce profound alterations in gut microbial ecology by rapidly reducing the abundance of obligate anaerobic commensals and overall microbial diversity. Human and animal studies have consistently demonstrated that ciprofloxacin treatment decreases members of the phyla *Firmicutes* and *Bacteroidetes* while promoting the expansion of Proteobacteria, particularly *Enterobacteriaceae*, resulting in reduced colonization resistance and ecological instability [39,40]. Ciprofloxacin exposure also enriches Antibiotic Resistance Genes (ARGs) and mobile genetic elements, including transposases, facilitating the expansion and persistence of resistant *Escherichia coli* populations within the intestinal resistome [41]. Furthermore, depletion of butyrate-producing bacteria such as *Faecalibacterium* and *Roseburia* reduces short-chain fatty acid production, compromises epithelial barrier integrity, and impairs regulatory immune pathways, thereby creating a pro-inflammatory intestinal environment that may promote IBD development in genetically susceptible individuals [32,42]. Because fluoroquinolones carry high IBD risk, other narrow-spectrum alternatives for common infections can be considered and microbiome-sparing measures can be used (e.g., probiotic co-therapy).

##### Nitroimidazoles

The nitroimidazole class of antibacterials includes metronidazole and tinidazole. They have broad-spectrum activity against parasites, mycobacteria, and anaerobic Gram-positive and Gram-negative bacteria. The nitro group is important for its mode of action and inhibits DNA synthesis, leading to DNA degradation and cell death [43].

Among this class, metronidazole was strongly associated with the risk of IBD [9]. Faye et al. (2023) described nitroimidazoles as the second highest-risk class (IRR 1.61, 95% CI 1.41–1.83), particularly among adults ≥60 years [22]. In children, repeated or early metronidazole use likewise elevates IBD risk [10,23]. According to a meta-analysis, the use of antibiotics in children, specifically metronidazole, was associated with an increased risk of inflammatory bowel disease (IBD), particularly CD (OR: 5.01; 95% CI: 1.65–15.25) [44]. Jakobsson et al. (2010) reported a lower diversity of the gut microbiota in throat and fecal samples immediately after metronidazole treatment as well as long-term effects on the gut microbiota [45]. Another study reported that the numbers of culturable *Bifidobacterium*, *Clostridium*, and *Bacteroides* spp. significantly decreased in feces after treatment, while the numbers of enterococci significantly increased one week after treatment. They also detected a persistent decrease in *Bifidobacterium* sp. and *Bacteroides* sp. 4 weeks after treatment [46]. Because metronidazole specifically targets obligate anaerobes, prolonged therapy may preferentially eliminate butyrate-producing commensals while allowing expansion of facultative anaerobic pathobionts, thereby compromising epithelial barrier integrity and promoting mucosal immune activation [32,42]. 

#### 2.3.2. Intermediate Risk Association Antibiotics

##### Macrolides

Macrolides are broad-spectrum antibiotics that inhibit bacterial protein synthesis, which in turn affects bacterial growth and replication. Due to their broad-spectrum nature, they affect both harmful and beneficial bacteria, resulting in a significant impact on the overall microbial community within the gastrointestinal tract [47].

Theochari et al., Scharf & Schlattmann (2024) also describe the risk associated with macrolide use in IBD and described a moderate risk of developing IBD but not as high a risk compared to nitroimidazoles and fluoroquinolones [48]. Individuals aged 40 years and older showed a higher association than any other age group (IRR 1.31, 95% CI 1.23–1.38) [22]. For pediatric IBD, Ungaro et al. 2014 performed a meta-analysis investigating antibiotic exposure as a risk factor for developing IBD and determined that macrolide use has a moderate risk association with new-onset IBD with a pooled OR of 1.231 (1.112–1.363 at 95% CI) [9].

Macrolides induce significant alterations in the intestinal microbiome despite their primary use for extraintestinal infections. Human studies have demonstrated that azithromycin treatment reduces the abundance of beneficial Actinobacteria, particularly *Bifidobacterium*, together with several Firmicutes, while promoting expansion of Proteobacteria and antibiotic-resistance determinants [49,50]. Macrolide exposure has also been associated with an increased risk of *Clostridioides difficile* infection, reflecting disruption of anaerobic colonization resistance and depletion of commensal bacteria that normally suppress pathogen overgrowth [51].

##### β-Lactams

Penicillins and cephalosporins come under the β-lactam antibiotic class. They have broad-spectrum activity against both Gram-positive and Gram-negative bacteria. β-Lactam antibiotics interfere with the synthesis of peptidoglycan and block transpeptidation activity, weakening the cell wall and increasing susceptibility to osmotic stress, ultimately leading to autolysis [52].

Cephalosporin was associated with the development of IBD, particularly CD [53]. Penicillin was associated with an increased risk for developing CD, but for UC it showed only a weak association [40]. Faye et al. (2023) showed that the risk for developing IBD was lower for narrow-spectrum penicillins (IRR 1.24, 95% CI 1.19–1.29) [22].

For both narrow-spectrum penicillin and extended-spectrum penicillin, persons aged 40 years and older showed the strongest association. Extended-spectrum penicillin has a relatively stronger association than narrow-spectrum penicillin [22].

β-Lactam antibiotics exert broad ecological effects on the intestinal microbiome owing to their extensive activity against Gram-positive and, in some cases, Gram-negative bacteria. A shotgun metagenomics study of children receiving macrolides or penicillins (β-lactams) found decreased microbial diversity and enriched ARG carriage from antibiotic exposure [41]. β-Lactam exposure was found to promote enrichment of β-lactamase genes and other antimicrobial resistance determinants within the gut resistome, contributing to long-term ecological instability and reduced colonization resistance [41]. β-lactam use was also found to be associated with enrichment of Extended-spectrum β-lactamases (ESBLs) harboring *E. coli* strains. IBD patients colonized intestinally with ESBL-producing isolates may have a more severe disease course [54].

#### 2.3.3. Low-Risk Antibiotics and Conflicting Associations

##### Tetracyclines

Tetracyclines are broad-spectrum antibiotics that include tetracycline hydrochloride (HCl), doxycycline, and minocycline [55]. Tetracyclines have a broad-spectrum of activity against both Gram-positive and Gram-negative bacteria. They exhibit antimicrobial activity by binding to the 30S ribosomal subunit, preventing the binding of aminoacyl-tRNA to the ribosome and consequently inhibiting bacterial protein synthesis [56].

Tetracycline use showed a moderate association with IBD (IRR, 1.35, 95% CI 1.21–1.50) [22]. Whereas other studies showed that there was no significant association between tetracycline use and IBD [57]. Similarly, Theochari et al. (2018) and Smaha & Money (2024) also report conflicting evidence with respect to tetracycline use and IBD risk [53,58].

Tetracyclines exhibit a highly nuanced clinical and ecological signature within the gastrointestinal tract. While they alter the gut flora by suppressing susceptible Gram-negatives and strict obligate anaerobes [59]. They also chelate dietary minerals such as Ca^2+^, Mg^2+^, and Zn^2+^ impacting absorption [60]. Mechanistic studies have shown shifts in gut taxa (e.g., reduction in Bacteroidetes) after tetracycline use [59]. Paradoxically, however, tetracyclines possess well-documented non-antimicrobial immunomodulatory properties by inhibiting host matrix metalloproteinases (MMPs), promoting innate immune responses and inducing mucosal healing [61]. This intrinsic anti-inflammatory mechanism may fundamentally offset their induced dysbiosis, explaining why large-scale epidemiological studies frequently report a more neutral or lower correlation with new-onset IBD for tetracyclines compared to other broad-spectrum antimicrobial classes.

##### Nitrofurans

Nitrofurantoin comes under the nitrofuran family and is used to treat urinary tract infections. It has activity against both Gram-positive and Gram-negative bacteria [62] and does not affect bowel flora [63]. Faye et al. (2023) showed nitrofurantoin was not associated with the development of IBD across all age groups, as it has less impact on gastrointestinal flora [22]. It does not significantly alter gut diversity or metabolic profiles. Thus, its use for cystitis or prophylaxis is not expected to perturb the gut microbiome or immune homeostasis.

#### 2.3.4. Other Antibiotics

Other classes include clindamycin (a lincosamide), sulfonamides (e.g., trimethoprim-sulfamethoxazole), and occasional use of drugs like chloramphenicol or colistin. Among these less frequently investigated antibiotic classes, clindamycin deserves particular attention because of its profound effects on the intestinal microbiome and its emerging association with inflammatory bowel disease (IBD). Clindamycin is a lincosamide antibiotic that inhibits bacterial protein synthesis and exhibits potent activity against anaerobic bacteria and many Gram-positive organisms. It is consequently commonly prescribed for skin and soft-tissue infections, dental infections, and acne management. However, Clindamycin is also known to cause severe gut dysbiosis and *C. difficile* infection (CDI). This is particularly relevant as patients with IBD have an increased risk of *Clostridioides difficile* infection (CDI) compared with those without IBD which is worsened by antibiotic use. CDI is a major inciting factor in IBD exacerbation and a common complication of IBD and the increased risk of CDI in the IBD population was attributable to the clindamycin and metronidazole classes of antibiotics [64].

Sulfonamides are a broad-spectrum class of antibiotics with activity against Gram-positive and certain Gram-negative bacteria. They are used for the treatment of certain bacterial infections, and they inhibit bacterial growth by preventing folate synthesis [65]. Faye et al. (2023) identified that for sulfonamides, the risk association was highest for ages 40–60, they deduced that for younger individuals, there is no significant association in developing IBD, but for older age groups, there is a slight increase in risk of IBD (age 40–60—IRR 1.19, 95% CI 1.08–1.31) [22]. However, Scharf et al. (2025) conducted a meta-analysis that revealed sulfonamides have no significant association in developing IBD [48].

Collectively, the available evidence indicates that antibiotic-associated IBD risk is strongly influenced by the antibiotic class and ecological impact on the gut microbiota. Classes that significantly deplete obligate anaerobes and short-chain-fatty-acid-producing commensals, including fluoroquinolones and nitroimidazoles, generally exhibit the strongest associations with IBD. In contrast, agents with limited gastrointestinal exposure, such as nitrofurantoin, show little or no increase in risk [22,23,59]. These observations support a microbiome-mediated model in which the magnitude and persistence of antibiotic-induced dysbiosis are key determinants of disease susceptibility. Although these associations are consistent across several large observational studies, they should be interpreted cautiously because confounding by indication, reverse causation, and differences in antibiotic prescribing practices may partially influence the observed risk estimates.

**Table 1:** Class-Specific Associations Between Antibiotic Exposure and Risk of Inflammatory Bowel Disease (IBD).

Antibiotic Class	Typical Uses	Most Affected Age Group	IRR (95% CI)	Overall Association in Developing IBD
Tetracyclines	Acne, rickettsial infections	40–60	1.35 (1.21–1.50)	Conflicting data [22,53,61]
Fluoroquinolones	Urinary Tract Infections (UTIs), GI infections, pneumonia	40–60	1.79 (1.61–1.97)	Highest risk [22]
Nitroimidazole	Anaerobic infections, *C. difficile*, Crohn’s perianal disease	60 and above	1.61 (1.41–1.83)	Highest risk [22]
Macrolides	Respiratory, *H. pylori*, some skin infections	40–60	1.31 (1.23–1.38)	Moderate risk [22]
β-Lactams	Respiratory, skin, GI infections (e.g., amox-clav, cephalosporins)	40–60	1.24 (1.19–1.29)	Weakest association [22]
Nitrofurantoin	Uncomplicated UTIs	-		Not associated [22]
Sulfonamides	UTIs, respiratory infections, Pneumocystis Pneumonia (PCP)	40–60	1.19 (1.08–1.31)	moderate risk [22,48]
Lincosamides (clindamycin)	skin and soft tissue infections	-	increased risk of CDI in IBD patients attributed to clindamycin exposure 0R 4.7 (1.9–11.9)	High risk of CDI [64]

## 3. Antibiotic-Induced Dysbiosis in IBD

The intestinal microbiome plays a central role in maintaining mucosal homeostasis through regulation of epithelial barrier integrity, immune tolerance, nutrient metabolism, and colonization resistance against pathogenic microorganisms [42,66]. In healthy individuals, this complex microbial ecosystem exists in a state of dynamic equilibrium with the host immune system. Disruption of this balance, commonly referred to as dysbiosis, is now recognized as a fundamental feature of inflammatory bowel disease (IBD), with both Crohn’s disease and ulcerative colitis exhibiting reduced microbial diversity, depletion of beneficial commensal bacteria, and expansion of pro-inflammatory pathobionts [66,67]. Antibiotic exposure induces rapid and persistent alterations in gut microbial composition, characterized by reduced diversity, depletion of beneficial commensals, and expansion of opportunistic taxa [68]. In a *Nature Medicine* cohort of nearly 15,000 individuals, Baldanzi et al. (2026) [12] reported that a single course of clindamycin within a year was associated with an average loss of ~47 bacterial species. One course of fluoroquinolone or flucloxacillin reduced species richness by ~20 each [12]. Over time, even antibiotics taken 1–4 years prior were linked to diminished diversity. The classes causing the largest disruptions were anti-anaerobes: clindamycin, broad-spectrum penicillins (e.g., amoxicillin-clavulanate), and fluoroquinolones [12]. Ciprofloxacin, despite its relatively low activity against standard cultivated anaerobes, profoundly alters gut microbiota. composition. Five days of ciprofloxacin influence about one-third of bacterial taxa in the gut and decrease taxonomic richness within a few days of initial exposure [13].

This susceptibility is highly dependent on the life stage at which exposure occurs, with the early-life window representing a critical period of vulnerability. During infancy and early childhood, the gut microbiota is immature, highly dynamic, and sensitive to environmental perturbations [69,70]. Early-life microbial colonization plays a vital role in training the innate and adaptive immune systems, regulating epithelial barrier integrity, and establishing immunological tolerance to commensal antigens. Systemic antibiotic exposure during this critical period disrupts this developmental program, predisposing the host to aberrant immune responses and chronic intestinal inflammation later in life [69,70,71].

### 3.1. Loss of Protective Commensals, Pathobiont Expansion, and Altered Microbial Metabolome

**Figure 2:**
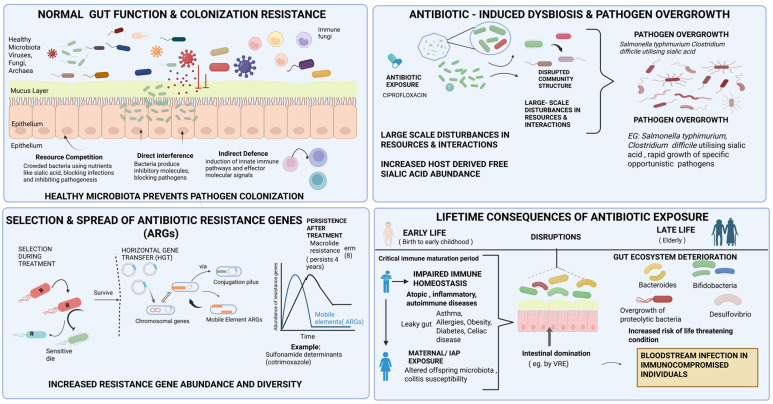
**Impacts of Antibiotic use on Gut Microbiome and its implications for host health and inflammatory bowel disease (IBD).** Antibiotic exposure disrupts the intestinal microbial ecosystem by reducing microbial diversity and depleting beneficial commensal bacteria, particularly short-chain fatty acid (SCFA)-producing taxa such as *Faecalibacterium*, *Roseburia*, and *Bifidobacterium*. These changes promote expansion of opportunistic pathobionts, including members of the Enterobacteriaceae, alter microbial metabolite production, impair epithelial barrier integrity, and reduce colonization resistance. Concurrent enrichment of antibiotic resistance genes further compromises microbiome resilience. Overall, these ecological and functional alterations promote immune dysregulation, chronic intestinal inflammation, and increased susceptibility to IBD development, disease progression, and recurrent flares. Created in BioRender. Nair, B. (2026) https://BioRender.com/phicwoe (accessed on 20 May 2026).

One of the earliest and most consistent consequences of antibiotic exposure is the disruption of microbial community structure, characterized by depletion of beneficial commensal microorganisms and expansion of opportunistic pathobionts. Although these alterations may occur transiently following a single antibiotic course, repeated or broad-spectrum antibiotic exposure can produce persistent ecological shifts that closely resemble the dysbiotic signatures observed in inflammatory bowel disease (IBD). Hallmarks of antibiotic-induced dysbiosis include reduced microbial diversity, depletion of obligate anaerobic bacteria belonging to the phyla Firmicutes and Bifidobacteria, and expansion of Proteobacteria, particularly members of the Enterobacteriaceae, a microbial pattern consistently associated with both Crohn’s disease and ulcerative colitis (Figure 2). The loss of protective commensals has important functional consequences for intestinal homeostasis. Several beneficial taxa, including *Faecalibacterium prausnitzii*, *Roseburia* spp., and *Bifidobacterium* spp., are major producers of short-chain fatty acids (SCFAs), particularly butyrate, which serves as the primary energy source for colonocytes and plays a critical role in maintaining epithelial barrier integrity, stimulating mucus production, promoting regulatory T-cell differentiation, and suppressing excessive inflammatory responses [72]. Antibiotic-mediated depletion of these organisms therefore results not only in taxonomic alterations but also in profound metabolic dysfunction that compromises mucosal immune homeostasis. Accordingly, antibiotic exposure consistently disrupts microbial metabolism by reducing the production of beneficial microbial metabolites. Across both animal models and human studies, broad-spectrum antibiotics have been shown to markedly decrease luminal concentrations of SCFAs, particularly butyrate. For example, Chang et al. (2018) treated mice with a broad-spectrum antibiotic cocktail and found luminal butyrate was reduced to nearly undetectable levels, with acetate and propionate also markedly decreased [3]. Similarly, pediatric patients undergoing hematopoietic stem cell transplant (HSCT) showed progressive declines in fecal butyrate and other SCFAs after broad-spectrum antibiotic exposure [73]. In preterm infants, detailed metabolomics revealed that antibiotic regimens disrupted gut metabolite profiles, notably depleting SCFAs and altering bile acids [74]. Metabolomic analyses consistently demonstrate that antibiotic exposure depletes key anti-inflammatory metabolites, including butyrate and other anti-inflammatory metabolites, in infants [75]. Comparable alterations have also been observed in adults, where anti-anaerobic antibiotic therapy resulted in significant reductions in circulating and fecal microbial metabolites, including SCFAs, indole derivatives, and cresol metabolites that normally contribute to epithelial barrier maintenance and immune regulation [47].

Beyond SCFA depletion, antibiotic-induced dysbiosis also promotes expansion of facultative anaerobic pathobionts. Loss of colonization resistance permits the overgrowth of members of the *Enterobacteriaceae*, including adherent-invasive *Escherichia coli* (AIEC), together with other opportunistic bacteria that thrive in the inflamed intestine [76,77,78]. Expansion of these bacteria increases exposure to lipopolysaccharide (LPS) and other microbe-associated molecular patterns (MAMPs), which activate pattern-recognition receptors such as Toll-like receptors (TLRs) and nucleotide-binding oligomerization domain-containing protein 2 (NOD2), leading to activation of NF-κB signaling and increased production of pro-inflammatory cytokines, including TNF-α, IL-6, and IL-23 [16,79]. In genetically susceptible individuals carrying variants in genes such as *NOD2*, *ATG16L1*, and *IL23R*, defective microbial sensing, impaired autophagy, and reduced bacterial clearance may further amplify these inflammatory responses, facilitating chronic intestinal inflammation characteristic of IBD [16,80].

Taken together, these findings indicate that the consequences of antibiotic exposure extend far beyond transient alterations in microbial composition. By simultaneously depleting beneficial commensals, reducing production of immunoregulatory microbial metabolites, and promoting expansion of inflammatory pathobionts, antibiotics shift the intestinal ecosystem toward a less resilient and more pro-inflammatory state. These ecological and functional disturbances closely mirror the microbial signatures observed in IBD, providing a compelling mechanistic explanation for the class-specific epidemiological associations discussed in the previous section.

### 3.2. Barrier Dysfunction, Colonization Resistance and Resistome Expansion

#### 3.2.1. Barrier Dysfunction

The intestinal epithelial barrier constitutes the first line of defence against luminal microorganisms and depends on the coordinated function of the mucus layer, antimicrobial peptides, and intercellular junctional complexes, including tight junctions, adherens junctions, and desmosomes [81,82]. Following antibiotic-induced dysbiosis, reduced availability of microbial metabolites, particularly butyrate and other short-chain fatty acids (SCFAs), together with a pro-inflammatory mucosal environment, compromises epithelial barrier integrity. Increased expression of pro-inflammatory cytokines, including TNF-a and IFN-γ, promotes activation of myosin light chain kinase (MLCK), leading to cytoskeletal remodelling and disruption of tight junction proteins such as occludins, claudins, and zonula occludens-1 (ZO-1), thereby increasing paracellular permeability [81,82]. Barrier integrity is further compromised by enhanced intestinal epithelial cell apoptosis and epithelial shedding, resulting in microerosions that impair mucosal repair and facilitate microbial translocation [81]. Concurrently, expansion of opportunistic pathobionts following antibiotic exposure exacerbates epithelial injury. Adherent-invasive *Escherichia coli* (AIEC) disrupts epithelial mitochondrial homeostasis, whereas enterotoxigenic *Bacteroides fragilis* (ETBF) secretes *B. fragilis* toxin (BFT), which cleaves E-cadherin and disrupts epithelial barrier architecture [82,83]. Collectively, these structural and functional defects increase intestinal permeability, allowing luminal microorganisms and microbial products to access the underlying mucosa, thereby promoting chronic intestinal inflammation characteristic of IBD [81,82].

#### 3.2.2. Loss of Colonization Resistance

Loss of epithelial integrity is accompanied by disruption of colonization resistance, a fundamental ecological function through which the resident microbiota prevents pathogen establishment. Under physiological conditions, commensal microorganisms occupy ecological niches, compete for essential nutrients such as carbohydrates and iron, and produce antimicrobial compounds including bacteriocins and microcins that suppress invading pathogens [84,85]. Resident microbiota also reinforce host defenses by stimulating production of secretory IgA, antimicrobial lectins, and other protective factors that further restrict pathogen colonization [84]. Antibiotic-mediated depletion of these keystone microorganisms, therefore, abolishes both microbial competition and host-mediated colonization resistance, creating ecological niches that favor opportunistic microorganisms and multidrug-resistant pathobionts [85,86].

Consequently, opportunistic pathogens rapidly exploit the disturbed intestinal environment and newly available metabolic substrates generated following loss of colonization resistance. Broad-spectrum antibiotic exposure has been associated with depletion of beneficial genera, including *Prevotella*, *Bifidobacterium*, and *Faecalibacterium*, accompanied by expansion of organisms such as *Enterococcus faecium*, *Streptococcus*, and multidrug-resistant Enterobacteriaceae [14,87]. Several of these pathobionts possess specialized metabolic adaptations that promote persistence within the inflamed intestine. For example, *Clostridioides difficile* utilizes succinate that accumulates following depletion of commensal Bacteroidetes, thereby enhancing its growth and toxin production [85]. Likewise, *Salmonella enterica* serovar Typhimurium exploits host-derived nutrients, including ethanolamine and fructose-asparagine, while employing virulence factors to invade the compromised intestinal epithelium [84,85]. Expansion of these opportunistic microorganisms further amplifies epithelial injury and microbial translocation, reinforcing the chronic inflammatory environment associated with IBD [82,85].

#### 3.2.3. Resistome Expansion

The ecological disruption created by barrier failure and loss of colonization resistance also establishes conditions that favor expansion of the gut resistome. Under the selective pressure imposed by antibiotic therapy, microorganisms carrying antibiotic resistance genes (ARGs) gain a competitive advantage, leading to enrichment of resistant taxa and long-term restructuring of the microbial community [14,88,89]. Simultaneously, the gut microbiota serves as a major reservoir of ARGs, facilitating dissemination of resistance determinants through horizontal gene transfer mediated by mobile genetic elements, including plasmids, transposons, and bacteriophages [14,15]. These processes collectively promote expansion of resistance genes conferring resistance to multiple antibiotic classes, including macrolides, aminoglycosides, tetracyclines, and glycopeptides [14,88,89].

In addition, broad-spectrum antibiotic treatment has been associated with marked alterations in the gut resistome, with 1955 antimicrobial resistance genes identified, of which only 159 were shared between antibiotic-treated individuals and healthy controls, indicating substantial restructuring of resistance profiles [88] (Figure 2). Resistance genes associated with glycopeptides, tetracyclines, aminoglycosides, and macrolides were frequently detected, while specific determinants including tetM, tet45, vanHM, and vanYM showed increased abundance following antibiotic exposure [88]. Increased transposase activity, together with enrichment of ABC transporters, further suggested enhanced horizontal gene transfer, mobilization of resistance determinants, and dissemination of antibiotic resistance mechanisms [88].

Importantly, resistome expansion frequently persists beyond completion of antibiotic therapy. Although microbial composition may partially recover, antibiotic exposure can leave a prolonged genetic imprint, often referred to as “antibiotic scarring,” characterized by sustained enrichment of ARGs despite incomplete restoration of the microbial community [86]. Persistence of resistance determinants such as the macrolide resistance gene erm(B) and tetracycline resistance genes (tetM and tetO) for months or even years after treatment illustrates the long-term stability of these genetic reservoirs [45,86,90]. Continued maintenance of ARGs within resident microbial communities may reduce microbiome resilience, facilitate recurrent colonization by multidrug-resistant pathobionts, and complicate restoration of intestinal homeostasis following antibiotic exposure [15,86]. Within the context of IBD, persistence and dissemination of ARGs may enhance survival of opportunistic microorganisms within the chronically inflamed intestine, while repeated antibiotic exposure during disease management may further enrich resistant microbial populations and promote dissemination of clinically important resistance determinants [15,88]. These findings indicate that resistome expansion represents not only a microbial adaptation to antibiotic exposure but also a mechanism that may perpetuate dysbiosis, sustain chronic intestinal inflammation, and increase therapeutic challenges in patients with IBD [14,15,88]. The different impacts of antibiotic use on gut microbiome and health of the host are summarized in Figure 2.

### 3.3. Microbiome Recovery, Resilience and Functional Recovery

**Figure 3:**
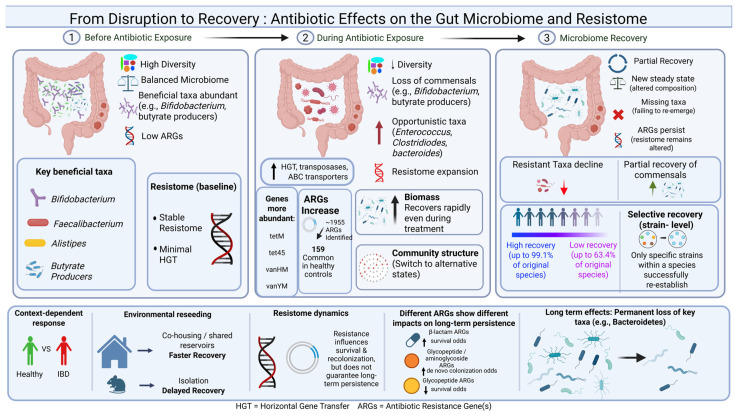
**Microbiome Disruption, Resistome Expansion, and Recovery Dynamics Following Antibiotic Exposure.** Antibiotic exposure rapidly disrupts the intestinal microbial ecosystem by reducing microbial diversity, depleting beneficial commensal bacteria, and promoting expansion of opportunistic pathobionts together with enrichment of the intestinal resistome. Although partial recovery of microbial diversity may occur following antibiotic withdrawal, restoration of the original microbial community and its metabolic functions is frequently incomplete. Recovery is influenced by factors including antibiotic class, duration of exposure, host age, baseline microbiome composition, diet, environmental microbial reseeding, and antimicrobial resistance. Consequently, repeated or broad-spectrum antibiotic exposure may result in a reorganized microbial ecosystem characterized by reduced resilience, persistent dysbiosis, altered microbial metabolism, and increased susceptibility to intestinal inflammation. These findings highlight the importance of microbiome-preserving and microbiome-restorative strategies to mitigate the long-term consequences of antibiotic therapy in patients with inflammatory bowel disease (IBD). Created in BioRender. Nair, B. (2026) https://BioRender.com/tqhftlj (accessed on 20 May 2026).

Antibiotic exposure in IBD patients induces substantial alterations in gut microbiome composition, with reduced diversity and decreased abundance of beneficial bacteria, particularly short-chain fatty acid-producing taxa. Following antibiotic treatment, the microbiome has demonstrated self-recovery capabilities, with most microbial communities recovering within 30 days. However, the microbiome still does not return to the pre-treatment baseline; instead, it reaches a new steady state different from the original composition. This equilibrium post-antibiotic treatment is characterized by reduced network complexity, indicating decreased microbial interactions, and increased prevalence of antibiotic resistance genes across multiple classes, with intra-phylum horizontal gene transfer occurring predominantly between taxonomically close organisms (Figure 3) [88]. All of this may compromise long-term microbiome resilience and increase dysbiosis in inflammatory bowel disease (IBD) patients. Longitudinal human studies consistently demonstrate that antibiotic-induced dysbiosis can persist well beyond the treatment period. Palleja et al. (2018) reported that although the gut microbiome gradually recovered following short-course broad-spectrum antibiotic therapy, several beneficial bacterial species failed to return even six months after treatment, indicating that ecosystem-level resilience may coexist with persistent taxonomic alterations [41]. Experimental studies provide mechanistic insight into these observations. Murine studies show similar patterns of disruption and incomplete recovery. Antibiotic administration caused a dramatic reduction in gut bacterial density within hours. However, total microbial biomass recovered rapidly to near pre-treatment levels, even during ongoing antibiotic exposure. This rapid rebound occurred alongside substantial shifts in community structure, indicating that restoration of microbial load does not necessarily reflect recovery of the original microbial composition. Furthermore, microbiome recovery followed distinct ecological trajectories rather than simply reversing the path of collapse [41,91]. Although higher-order taxonomic composition may appear to normalize, species- and strain-level diversity frequently remains reduced, with selective loss of key commensals, particularly members of the Bacteroidetes phylum. These findings suggest that apparent taxonomic recovery may mask substantial ecological simplification and loss of functional redundancy.

Microbiome resilience is further influenced by strain-specific characteristics, host factors, and environmental microbial exposure. High-resolution metagenomic analyses have shown that only selected strains within individual commensal species successfully recolonize following antibiotic treatment, whereas others remain permanently depleted. Similarly, co-housing experiments in mice demonstrated that environmental microbial reservoirs facilitate recolonization of beneficial taxa, whereas microbial isolation delays recovery and promotes long-term dysbiosis [41,91]. These observations indicate that recovery depends not only on surviving bacteria but also on opportunities for microbial reseeding and restoration of ecological interactions (Figure 3).

A prospective study examining four commonly prescribed antibiotics reported an acute reduction in bacterial load and species richness. Although apparent recovery occurred within approximately two months, persistent alterations in microbial composition, resistome profiles, and metabolic function were observed, indicating incomplete restoration of the original microbiome state. Recovery also varied among individuals, with some participants recovering only 63.4% of their original microbial species compared with 99.1% recovery in others. In some individuals, microbiome profiles shifted toward patterns resembling those observed in critically ill patients, indicating the potential for persistent dysbiosis following antibiotic exposure [86]. Reduced microbial diversity, depletion of short-chain fatty acid-producing bacteria and altered taxonomic composition have been reported following antibiotic exposure, contributing to persistent dysbiosis and impaired microbiome resilience. Functional recovery may also remain incomplete even when microbial composition appears to normalize, further emphasizing the long-term consequences of antibiotic-induced microbiome disruption [92].

From an IBD perspective, incomplete microbiome recovery may have important clinical consequences. Persistent depletion of short-chain fatty acid-producing bacteria together with expansion of pathobionts and antibiotic resistance genes creates a less resilient microbial ecosystem that may sustain low-grade mucosal inflammation and increase susceptibility to disease relapse. These findings suggest that repeated antibiotic exposure may progressively lower the threshold for chronic intestinal inflammation in genetically susceptible individuals, emphasizing the importance of antimicrobial stewardship and microbiome-restorative strategies in IBD management. The major stages of antibiotic-induced dysbiosis, resistome expansion, and microbiome recovery are summarized in Figure 3.

## 4. Strategies to Reduce Antibiotic-Associated Risk in IBD

### 4.1. Prevention of Antibiotic-Induced Microbiome Disruption

During antibiotic administration, the fraction of the drug that is not absorbed, along with residues processed through the bile, makes its way to the cecum and colon, where it can significantly affect the resident microbial communities [93]. These changes allow opportunistic pathogens to emerge and drive the expansion of antibiotic-resistant microorganisms. During antibiotic exposure, alongside the depletion of beneficial commensal bacteria such as *Faecalibacterium prausnitzii*—which is directly linked with CD—antibiotics are also shown to induce an outgrowth of potentially pathogenic species such as *Bacteroides vulgatus*, *Parabacteroides distasonis*, and *Fusobacterium varium*, which have been linked to the development of colitis in both animal models and clinical studies [94].

Various strategies have been developed to limit the microbiome disruption caused by antibiotics reaching the cecum and colon while ensuring that systemic antibiotic activity is preserved [93]. One such preventative strategy against antibiotic-induced dysbiosis is to degrade unabsorbed antibiotics in the upper gastrointestinal tract with the administration of β-lactamase enzymes before they can harm the colonic microbiome. SYN-004 (ribaxamase) is an engineered β-lactamase developed to degrade intravenous cephalosporins like ceftriaxone. In porcine models, it successfully averted dysbiosis and significantly lowered the emergence of the gut resistome without interfering with the therapeutic efficacy of the drug [94]. Similarly, the recombinant class A β-lactamase that was delivered orally protected the colonic microbiota in mice models after the administration of ampicillin or piperacillin [95]. Additionally, the utilization of naturally occurring β-lactamase-producing anaerobes offers another alternative for the mitigation of ceftriaxone-induced damage in the gut, hence sustaining the integrity of intestinal colonization resistance and defending against microbial composition alterations [96].

While this strategy protects the gut microbiota without interfering with the systemic drug activity, its scope of application is limited due to its narrow spectrum, since it does not extend to other widely used antibiotics like fluoroquinolones [93].

Other strategies, such as non-enzymatic inactivation using targeted adsorbents like activated charcoal encapsulated within zinc-pectinate beads, can selectively deplete the residual fraction of fluoroquinolones like ciprofloxacin through adsorption within the lower gastrointestinal tract. This approach protects the gut microbial ecosystem and overcomes previously addressed limitations due to its broad spectrum of activity [97].

A targeted charcoal delivery system, DAV132, was designed to release activated charcoal in the terminal ileum and colon; it has shown substantial clinical efficacy in both preclinical and clinical settings [93]. The clear advantage of this approach is evidenced by ex vivo analysis using pig caecal medium, where thirteen of fourteen tested antibiotics reached at least 95% adsorption. These findings are supported by preclinical data that demonstrated that such adsorbents significantly reduce intestinal antibiotic concentrations without affecting systemic drug levels. Similarly, human trials indicate that DAV132 reduces fecal antibiotic concentrations by up to 99%, thereby preventing long-term dysbiosis while maintaining excellent patient tolerability [93]. Although currently evaluated primarily in preclinical studies and early clinical trials, these approaches offer considerable promise for reducing antibiotic-associated dysbiosis and may ultimately help lower the risk of IBD development and disease exacerbation in susceptible individuals.

Future microbiome-preserving strategies are likely to extend beyond enzymatic degradation and intestinal adsorption toward precision approaches tailored to individual patients. Integration of microbiome profiling, pharmacokinetic modeling, and antibiotic stewardship may enable clinicians to identify patients at highest risk of antibiotic-associated dysbiosis and implement targeted microbiome-protective interventions. Such precision approaches could be particularly valuable for genetically susceptible individuals or patients with established IBD who require repeated antibiotic therapy.

### 4.2. Microbiome Restoration Strategies

Although microbiome-preserving strategies can reduce collateral damage during antibiotic therapy, they are unable to fully reverse established dysbiosis once it has occurred. Because repeated antibiotic exposure in inflammatory bowel disease (IBD) patients often results in incomplete microbiome recovery and persistent functional alterations, increasing attention has focused on therapeutic approaches that actively restore microbial diversity, metabolic function, and intestinal immune homeostasis. Among these Fecal Microbiota Transplantation (FMT) stands as a major tool for this purpose. The procedure starts by transferring a well-screened and minimally processed stool from a healthy donor into the gastrointestinal tract of the patient. The goal of this intervention is to restore microbial balance and increase microbiome diversity that may contribute to the attenuation of intestinal inflammation.

Clinical studies have demonstrated encouraging, although variable, outcomes following FMT in ulcerative colitis (UC). Meta-analyses report clinical and endoscopic remission rates of approximately 24–36% in patients with active UC, together with improvements in microbial diversity and a shift in recipient microbial composition toward donor-like communities [98]. Restoration of microbial richness is accompanied by important immunological changes, including reduced mucosal infiltration of CD8^+^ T cells, decreased expression of pro-inflammatory cytokines such as IL-6 and IP-10, and increased production of the regulatory cytokines IL-10 and TGF-β, collectively contributing to attenuation of intestinal inflammation [99].

In clinical settings, FMT has been shown to provide clinical benefits in the management of UC and CD. Endoscopic and clinical benefits were observed. FMT has been suggested as a means of preventing disease flares. However, in reality, it is still hard to be sure that the results will always be the same. Results can be affected by donor selection and the successful engraftment of microbiota. Treatment with antibiotics should also be planned according to recent antibiotic treatment, since antibiotic treatments are normally interrupted at least 24 h before transplantation to allow for microbial colonization. The procedure is generally well tolerated, and the majority of the side effects are mild and gastrointestinal. However, concerns remain regarding the risk of infection transmission and the need for more long-term safety data and standardized treatment protocols [99].

The success of treatment varies between individuals and is associated with differences in the microbial composition and their biological functions. Higher levels of beneficial bacteria are associated with successful procedures. Species such as *Eubacterium hallii*, *Roseburia inulinivorans*, and *Ruminococcus bromii* are enriched in these circumstances. Individuals with a different gut microbial profile are less likely to respond. In these cases, higher abundances of taxa such as *Fusobacterium*, *Sutterella*, and *Escherichia* strains are observed and are associated with non-response [100]. 

Microbial function also differs across taxonomy. Successful treatments improve metabolic processes related to gut homeostasis, such as the production of short-chain fatty acids (SCFA), the synthesis of secondary bile acids, and carbohydrate metabolism. Non-responders show a different functional profile with increased activity in pathways such as lipopolysaccharide and heme biosynthesis. Metabolomic analyses also provide further evidence for these differences, with differences in metabolites like lysine, heme, and bile acid derivatives depending on the treatment outcome, and then there is the donor’s biology. In general, higher levels of *Bacteroides* in the donor stool have been associated with positive outcomes in the recipient, while *Streptococcus* species have been associated with less favorable responses. The use of stool pools from multiple donors has been proposed to increase microbial diversity. The mixing of donor samples increases the baseline diversity of the microbiota and may contribute to improved treatment outcomes [100].

Researchers increasingly want to find new ways to change the gut microbiome. The main approaches are dietary change and direct introduction of microorganisms. Prebiotics, for instance, are certain types of dietary fibers that are not digested in the upper gastrointestinal tract. Rather, they are used as substrates by beneficial gut bacteria, producing short-chain fatty acids such as acetate, propionate, and butyrate. These molecules are important for the maintenance of the mucosal barrier. This metabolic process is also related to the regulation of the immune system and inhibition of pathogenic microorganisms. However, clinical results have been inconsistent when these prebiotic effects have been translated to patients with IBD.

Probiotics are an adjunctive approach. Clinicians give the patient live microorganisms directly. Once in the gut, these bacteria can out-compete pathogenic species and reinforce the intestinal barrier. The local environment produces antimicrobial substances and modifies the host immune response. These mechanisms are well described, but consistent clinical success is difficult to achieve as outcomes may depend on the specific bacterial strain used [101].

Synbiotics are formed by combining prebiotics and probiotics. The clinical goal is to increase the survival and activity of the introduced bacteria. Some studies have indicated that this combination method could reduce disease activity and inflammatory markers, but the present evidence is insufficient to recommend definitive clinical guidelines [101]. There are some risks involved in the use of live bacteria; hence scientists are putting their interest in paraprobiotics and postbiotics. These alternatives are thought to have more predictable effects. Paraprobiotics are non-viable microbial cells (intact or broken) or crude cell extracts that confer a benefit on the host by immunomodulation, without the risks of live microbes. The concept is extended to postbiotics through the direct use of microbial metabolites. Short-chain fatty acids and tryptophan derivatives are examples. These compounds play a role in the modulation of host immunity and supporting the intestinal barrier’s function. Butyrate plays a key role as a prominent energy source for colonocytes and as a stimulator of anti-inflammatory cytokine production and inhibition of pro-inflammatory pathways such as NF-κB. While these mechanisms are well characterized, there is variability in clinical responses observed in patients [101].

Among microbiome modulation strategies, probiotics have been most extensively investigated, warranting closer examination of their strain-specific and clinical effects. Experimental studies demonstrate that specific strains, particularly *Bifidobacterium* species, can reduce mucosal damage, inflammatory markers, and disease severity while improving epithelial integrity and cytokine profiles. Certain strains, such as *Bifidobacterium animalis* subsp. *lactis* and *Bifidobacterium longum* subsp. *infantis*, have also been shown to reduce epithelial apoptosis and pro-inflammatory cytokine production. Clinical studies report mixed but promising outcomes, with evidence supporting their role in maintaining remission in CD and sustaining remission in UC, particularly when multi-strain formulations are used alongside standard therapies. Nonetheless, variability in clinical responses reflects differences in strain composition, dosage, disease subtype, and individual patient factors [102].

### 4.3. Dietary and Emerging Microbiome-Targeted Strategies

**Figure 4:**
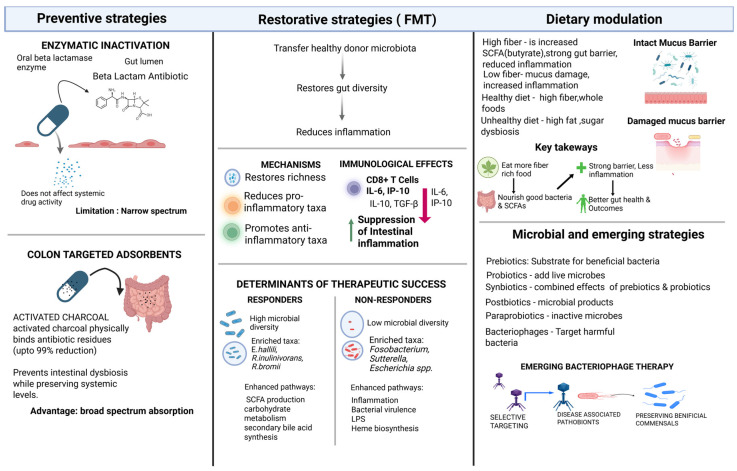
**Strategies to Reduce Antibiotic-Associated Risk and Restore Gut Microbiome Homeostasis in Inflammatory Bowel Disease.** Emerging strategies aim to minimize the detrimental effects of antibiotics on the gut microbiome while preserving their therapeutic efficacy. Microbiome-preserving approaches include enzymatic degradation of residual antibiotics (e.g., ribaxamase), targeted intestinal adsorbents (e.g., DAV132), and antimicrobial stewardship to reduce unnecessary broad-spectrum antibiotic exposure. Following antibiotic-induced dysbiosis, microbiome restoration strategies—including fecal microbiota transplantation (FMT), prebiotics, probiotics, synbiotics, paraprobiotics, and postbiotics—seek to re-establish microbial diversity, restore beneficial microbial metabolites, reinforce epithelial barrier integrity, and promote immune homeostasis. Together, these interventions represent complementary approaches for mitigating antibiotic-associated dysbiosis, improving microbiome resilience, and reducing disease progression and relapse in patients with IBD. (↑—increase; ↓—decrease). Created in BioRender. Nair, B. (2026) https://BioRender.com/y4tu700 (accessed on 20 May 2026).

Dietary factors play a central role in shaping the gut microbiome and represent a complementary strategy for mitigating dysbiosis associated with IBD. Diet directly influences microbial composition, diversity, and metabolic activity, thereby modulating host immune responses and intestinal homeostasis. While a balanced diet supports a diverse and stable microbial community, dietary imbalance can lead to dysbiosis, reduced microbial diversity, and impaired gut barrier function, all of which are implicated in IBD pathogenesis (Figure 4). Notably, antibiotic use further disrupts the commensal microbiome and reduces essential microbial metabolites, with recovery often being slow and incomplete, highlighting the importance of dietary interventions in microbiome restoration [103].

Among dietary components, fiber plays a particularly critical role in maintaining microbial diversity and intestinal barrier integrity. Dietary fiber serves as a substrate for microbial fermentation, leading to SCFA production, which supports epithelial integrity, immune regulation, and mucosal homeostasis. These metabolites also act as energy sources for colonocytes and enhance tight junction expression. In contrast, reduced fiber intake shifts microbial metabolism toward utilization of proteins and host-derived mucus, resulting in harmful metabolite production and increased inflammatory responses [103,104]. Fiber deficiency further promotes expansion of mucus-degrading bacteria, leading to erosion of the mucus barrier and increased susceptibility to inflammation, whereas adequate intake supports mucus production and maintains a functional intestinal barrier [103,104].

Beyond fiber, overall dietary patterns influence gut microbiota and inflammatory status. Diets high in fat reduce microbial diversity and promote pro-inflammatory bacteria, while high intake of animal protein increases production of metabolites such as hydrogen sulfide that impair epithelial integrity. Similarly, high sugar intake and food additives negatively affect microbial composition and barrier function. In contrast, low-fat, high-fiber dietary patterns are associated with reduced inflammation and improved clinical outcomes in IBD [103].

Bacteriophage therapy represents an emerging microbiome-targeted strategy with the potential to selectively modulate gut microbial composition in IBD (Figure 4). Bacteriophages are viruses that specifically infect bacteria and constitute an integral component of the gut microbiome, where they influence bacterial community structure and dynamics. In IBD, alterations in the gut microbiota are accompanied by shifts in the phage community, including increased phage abundance and disruption of phage–bacteria equilibrium, which may contribute to disease-associated dysbiosis and inflammation [105].

Therapeutically, exogenous administration of bacteriophages offers a highly targeted approach to eliminate disease-associated pathobionts while preserving beneficial commensal bacteria. Due to their high specificity, phages can selectively target bacterial strains at the species or even strain level, in contrast to broad-spectrum antimicrobial approaches that disrupt overall microbial diversity. Experimental evidence further demonstrates that phage combinations can effectively reduce pathogenic bacterial populations, such as *Klebsiella pneumoniae*, and attenuate inflammation in preclinical models. Mechanistically, phages reshape microbial communities through lytic activity and may also exert immunomodulatory effects, including induction of inflammatory responses such as interferon-γ production via toll-like receptor 9 signaling, although effects may vary depending on context.

Despite these promising features, several obstacles hamper the clinical implementation of phage therapy, including the emergence of phage-resistant bacterial mutants, difficulties in phage selection and delivery, possible off-target effects, and the necessity for accurate identification of disease-relevant bacterial targets. Regulatory constraints and a lack of standardized protocols further limit widespread use. Consequently, further research is required to establish safety, efficacy, and clinical applicability [105]. The major microbiome-preserving and microbiome-restorative and emerging strategies discussed in this section are summarized in Figure 4.

## 5. Conclusions and Future Perspectives

Antibiotics play a complex and paradoxical role in IBD, as both useful therapeutic agents and possible contributors to disease progression. Antibiotics are still used to treat certain complications of IBD, such as perianal disease, abscesses, pouchitis, and bacterial overgrowth. However, there is increasing evidence that exposure to antibiotics, particularly broad-spectrum and repeated courses, is associated with an increased risk of developing IBD and worsening of the disease. These effects seem to be very specific to the antibiotic class, duration of exposure, timing of administration and cumulative dose.

Substantial epidemiological evidence establishes antibiotic exposure—particularly during early life and older adulthood—as a significant environmental risk factor for new-onset IBD and acute disease flares. This risk is not uniform but is antibiotic class-specific. Broad-spectrum antibiotics, notably fluoroquinolones and nitroimidazoles, present the highest associations with IBD development. Mechanistically, these class-specific differences mirror their distinct disruptive capacities on the gut microbiome. High-risk agents drive profound, long-lasting dysbiosis marked by the taxonomic depletion of key commensal phyla (Firmicutes and Bacteroidetes) and the subsequent loss of vital anti-inflammatory metabolites like short-chain fatty acids (such as butyrate). This metabolic and taxonomic collapse compromises the mucosal barrier, giving rise to persistent immune dysregulation and expansion of pathobionts. Furthermore, a strong dose–response relationship has been identified, where increased cumulative exposure (three or more courses) further amplifies this risk trajectory.

While establishing absolute causality remains challenging due to inherent biases like reverse causation and confounding by indication, a “multi-hit” framework is widely supported. In this model, antibiotic-induced dysbiosis acts in tandem with genetic susceptibilities and other environmental triggers to fuel the pathogenesis of chronic intestinal inflammation.

To bridge the gap between antibiotic therapy and risk mitigation in IBD, future research and clinical practices must prioritize the following directions:

### 5.1. Longitudinal Multi-Omics Cohorts to Improve Causal Inference

Future observational and prospective studies should aim to better distinguish true antibiotic-driven IBD pathogenesis from confounding factors such as indication bias and reverse causation. Integrating longitudinal clinical monitoring with comprehensive multi-omics approaches—including metagenomics, metatranscriptomics, and metabolomics—may help define temporal changes in microbiome disruption and recovery while identifying key determinants and baseline thresholds associated with microbial resilience.

### 5.2. Defining Biomarkers of Vulnerability

Future research should move beyond viewing antibiotic-associated IBD risk as a uniform phenomenon and instead focus on identifying host and microbial biomarkers that influence individual susceptibility. Understanding how genetic variants associated with IBD susceptibility, such as *NOD2* and *ATG16L1*, interact with antibiotic-induced alterations in microbiome structure and function may enable risk stratification and support more personalized antibiotic prescribing strategies. Furthermore, integrative computational models combining antibiotic exposure history, longitudinal microbiome dynamics, and host genetic susceptibility could improve prediction of antibiotic-associated dysbiosis and facilitate precision antimicrobial stewardship in IBD.

### 5.3. Leveraging Artificial Intelligence and Systems Biology

Advances in artificial intelligence and systems biology offer new opportunities to integrate multidimensional clinical, microbiome, metabolomic, and genomic datasets into predictive models of antibiotic response. Machine learning approaches may facilitate identification of patient-specific microbial signatures associated with antibiotic susceptibility, dysbiosis resilience, and therapeutic response, ultimately supporting personalized antibiotic selection and microbiome-directed interventions in IBD.

### 5.4. Developing Microbial Co-Therapies

To preserve the gut microecology when antibiotic use is mandatory, future therapeutic regimens must investigate the simultaneous deployment of protective agents. Co-prescribing targeted probiotics (such as Bifidobacterium or VSL#3), tailored prebiotics, postbiotics, or synbiotics could shield keystone commensal taxa and maintain local SCFA and butyrate production during antibiotic administration.

### 5.5. Advancing Precision Antimicrobial Targeting

There is an increasing need to move away from broad-spectrum antibiotic therapies that cause extensive collateral disruption of commensal microbial communities toward more selective and targeted antimicrobial approaches. Future strategies may include the development of narrow-spectrum antibiotics, engineered bacteriophages, and small-molecule inhibitors directed against specific pathobionts, such as pathogenic *Escherichia coli* or members of the *Enterobacteriaceae*. Such targeted interventions may help preserve beneficial microbial networks while minimizing antibiotic-induced dysbiosis and its associated inflammatory consequences.

### 5.6. Implementing Antimicrobial Stewardship Frameworks

At a clinical and public health level, stronger antimicrobial stewardship strategies are essential to minimize unnecessary antibiotic exposure and reduce potential IBD-associated risks. Particular emphasis should be placed on pediatric populations and individuals presenting with early or subclinical gastrointestinal symptoms, where microbiome disruption may have long-term consequences. Restricting inappropriate broad-spectrum antibiotic use, limiting cumulative exposure, and prioritizing narrow-spectrum agents or non-oral routes of administration when possible, may help reduce microbiome disruption and lower population-level environmental triggers associated with IBD development.

Collectively, the evidence reviewed here supports a paradigm shift in the way antibiotics are viewed in inflammatory bowel disease. Beyond their role as antimicrobial agents, antibiotics should also be recognized as powerful ecological modulators capable of reshaping the intestinal microbiome, altering immune homeostasis, and influencing long-term disease susceptibility. Future integration of antimicrobial stewardship, precision antimicrobial therapies, microbiome-preserving technologies, systems-level modeling, and personalized microbiome restoration strategies offers the potential to maximize the therapeutic benefits of antibiotics while minimizing their unintended consequences. Ultimately, integrating antibiotic exposure history with host genetics, microbiome composition, and immune profiling will be fundamental to developing precision medicine approaches capable of predicting individual risk, guiding therapeutic decisions, and improving long-term outcomes for patients with IBD.

## Data Availability

No new data were created or analyzed in this study. Data sharing is not applicable to this article.

## Abbreviations

IBD	Inflammatory Bowel Disease
UC	Ulcerative Colitis
CD	Crohn’s Disease
FMT	Fecal Microbiota Transplantation
MAP	*Mycobacterium avium* subspecies *paratuberculosis*
IRR	Incidence Rate Ratio
CI	Confidence Interval
OR	Odds Ratio
GI	Gastrointestinal
HR	Hazard Ratio
SCFA/SCFAs	Short-Chain Fatty Acid(s)
ARG/ARGs	Antibiotic Resistance Gene(s)
ESBL/ESBLs	Extended-Spectrum β-Lactamase(s)
CDI	*Clostridioides difficile* Infection
UTIs	Urinary Tract Infections
PCP	Pneumocystis Pneumonia
HSCT	Hematopoietic Stem Cell Transplant(ation)
AIEC	Adherent-Invasive *Escherichia coli*
ETBF	Enterotoxigenic *Bacteroides fragilis*
BFT	*Bacteroides fragilis* Toxin
LPS	Lipopolysaccharide
MAMPs	Microbe-Associated Molecular Patterns
TLRs	Toll-Like Receptors
NOD2	Nucleotide-Binding Oligomerization Domain-Containing Protein 2
MLCK	Myosin Light Chain Kinase
ZO-1	Zonula Occludens-1
TNF-α	Tumor Necrosis Factor-α
IFN-γ	Interferon-γ
NF-κB	Nuclear Factor κB
IL-6	Interleukin-6
IL-10	Interleukin-10
IL-23	Interleukin-23
IP-10	Interferon Gamma-Induced Protein 10
TGF-β	Transforming Growth Factor-β
MMPs	Matrix Metalloproteinases
SYN-004	Ribaxamase (investigational β-lactamase)
VSL#3	Commercial multi-strain probiotic formulation
